# Aortic root ectasia as a phenotypic feature of a mitochondrial disorder

**DOI:** 10.1002/ccr3.1652

**Published:** 2018-06-17

**Authors:** Josef Finsterer

**Affiliations:** ^1^ Krankenanstalt Rudolfstiftung Vienna Austria

**Keywords:** aorta, metabolic defect, mitochondrial disorder, multisystem disorder, oxidative phosphorylation, vascular smooth muscle cells

## Abstract

Mitochondrial disorder (MID) can be suspected upon application of the mitochondrial multiorgan disorder syndrome score; aortic root ectasia (ARE) can be a phenotypic feature of MIDs; ARE in a MID may result from affection of vascular smooth muscle cells by the metabolic defect; ARE requires long‐term follow‐up not to miss the point at which ARE transforms to an aneurysm requiring vascular surgery.

## 1 INTRODUCTION

Involvement of the arteries is increasingly recognized as a rare phenotypic feature of mitochondrial disorders (MIDs).^1^ Arteriopathy in MIDs manifests as atherosclerosis, stenosis, occlusion, dissection, ectasia, aneurysm formation, spontaneous rupture, or arterio‐venous malformation.^1^ Mitochondrial arteriopathy may involve the cerebral, cervical, retinal, brachial, iliac, or muscular arteries, or the aorta.^1^ Recently, adult aortic root ectasia (ARE), defined as an aortic diameter of 40‐50 mm at the level of the aortic valves, has been described as a manifestation of a MID.^2^ Here, we report ARE as a vascular manifestation in a patient with nonspecific mitochondrial multiorgan disorder syndrome (MIMODS).

## 2 CASE REPORT

The patient is 84‐year‐old Caucasian woman, height 160 cm, weight 50 kg, who was referred for impaired consciousness. Her previous history was noteworthy for dementia, aphonia, hypothyroidism, strumectomy, hysterectomy, arterial hypertension, paroxysmal atrial fibrillation, lung emphysema, and diabetes. Clinical neurologic examination revealed sopor, positive frontal signs, rigor, cogwheel‐rigidity bilaterally, exaggerated tendon reflexes, and diffuse wasting on the upper limbs, and reduced tendon reflexes, positive pyramidal signs, diffuse wasting, and calf fasciculations on the lower limbs. Cerebral CT showed diffuse atrophy, basal ganglia calcification, and leucencephalopathy. Cerebral MRI revealed multiple, spot‐like, embolic, ischemic lesions, some microbleeds, diffuse cerebral atrophy, and leucencephalopathy. Electroencephalography was abnormal revealing a discontinuous, nonconvulsive epileptic state. Blood tests showed anemia, hyponatriemia, transient hypokaliemia, and a HbA1c of 6.4% (n, <6.0%). Resting serum lactate was increased to 2.4 mmol/L (n, <2.0 mmol/L). A previous lactate stress test was highly abnormal. Routine ECG showed an AV‐block III without indication of implantation of a pacemaker. Repeated X‐ray of the lung revealed ARE of 46 mm. ARE was confirmed by CT‐angiography of the aorta. Magnetic resonance imaging angiography was scheduled, but the patient received a DNR order and died during the further course before the investigation and work‐up for MID could have been carried out. Based upon the history, the clinical examination, the instrumental investigations, and the MIMODS score (n = 43) (see Appendix S1 ),^3^ MIMODS was diagnosed.

## 3 DISCUSSION

This case shows that ARE can be a phenotypic feature of a MID. This finding conforms with a recent study on 10 MID patients with ARE.^2^ In this study, the aortic root *Z*‐scores ranged between 2.1 and 3.6 (n: 0).^2^ One patient carried a single mtDNA deletion, 2 patients were diagnosed with mtDNA depletion, and in 3 patients, no mtDNA mutation was detected. In 3 further patients, no genetic studies had been carried out. The cause of ARE was attributed to involvement of the vascular smooth muscle cells (VSMCs) in the underlying metabolic defect.^2^ Whether ARE is associated with an increased risk of aortic dissection is unproven, but there are some indications that aortic root dilatation carries an increased risk of dissection and spontaneous rupture.^4^

The mechanism involved in the pathogenesis of ARE is unknown but may be similar to the pathogenesis of the more common aortic aneurysmal disease.^2^ Indications for a causal relation between MID and ARE are that arteriopathy affecting the intracerebral arteries (dilative arteriopathy, small vessel disease), the retinal arteries (LHON), the carotid arteries (carotid artery dissection. occlusion), the brachial arteries, the iliac arteries (Leriche syndrome), or the aorta (aortal rupture) has been previously reported as a phenotypic feature of a MID.^1^, ^5^, ^6^, ^7^ There are also indications that atherosclerosis is a primary manifestation of a vasculopathy in MIDs^8^ and that the cerebral arteries contribute to the pathogenesis of stroke‐like episodes in MELAS syndrome.^9^, ^10^ Long‐term implications of ARE, however, are currently incalculable.

Aortic root ectasia may not only be due to a mitochondrial defect but also due to arterial hypertension,^11^ Marfan syndrome,^12^, ^13^ osteogenesis imperfecta,^14^ senescence, or congenital heart defects (anulo‐aortic ectasia).^15^, ^16^ Additionally, ARE has been recently detected as a major determinant of aortic dilation.^17^ Presumably, atherosclerosis contributes to the development of ARE but, interestingly, ascending aorta aneurysms are associated with decreased systemic atherosclerosis.^18^ Whether ARE in the presented patient was simply due to her advanced age or truly a manifestation of the presumed underlying MID remains speculative, but the large number of abnormalities found in our patient and the high MIMODS score favor a mitochondrial metabolic defect to have been causative. The strongest argument against arterial hypertension or age as the causes of ARE is the multisystem nature of the clinical presentation. Although a MIMODS score of 43 is highly suggestive of a MID, the diagnosis needs to be confirmed by histological, immune‐histological, histochemical, ultrastructural, biochemical, or genetic data. In case these investigations are not available, determination of urine organic acids or of serum amino acids can be useful to screen patients for suspected MID. A further valuable screening test for MIDs is the lactate stress test.

In conclusion, this case shows that MID can be suspected upon application of the MIMODS score, that ARE can be a phenotypic feature of a MID. ARE in a MID may result from affection of the VSMCs by the underlying metabolic defect. Such patients need long‐term follow‐up not to miss the point at which ARE transforms to an aneurysm of the ascending aorta requiring vascular surgery. Whether ARE is prone to future aortic dissection remains speculative.

## AUTHOR CONTRIBUTION

JF: transformed the idea into the first draft, collected and read the literature, and conceptualized the final draft.

## CONFLICT OF INTEREST

None.
