# Supplementary material for: Aortic root ectasia as a phenotypic feature of a mitochondrial disorder
Source: Clin Case Rep. 2018 Jun 17;6(8):1501–3. doi: 10.1002/ccr3.1652 (PMC6099020; doi:10.1002/ccr3.1652)
Supplement: Supplementary file 1 [file CCR3-6-1501-s001.doc]

#### Appendix S1. MIMODS score

The MIMODS score is a clinical tool to assess upon phenotypic items and their frequency described in the literature if a patient’s clinical presentation is suggestive of a mitochondrial disorder (MID) or not. The score was generated from 36 definite MID patients diagnosed upon biochemical or genetic investigations. The score is composed of three elements. First, how many organs or tissues are affected by the metabolic defect. Second, how many abnormalities are found within all affected organs, and third, how frequent is a certain abnormality reported in the literature. If an abnormality achieves <50 hits, one point is awarded, if 51 to 100 are achieved, 2 points are awarded, and if >100 hits are achieved, 3 points are awarded. A MIMODS score >10 was calculated to suggest the presence of a MID. For the definite diagnosis of a MID further investigations, particularly, histological, immunehistological, histochemical, ultrastructural, biochemical, polarogrpahic, and genetic investigations, are inevitable.
